# Corneal Epithelial Damage and Impaired Tear Functions in Patients with Inflamed Pinguecula

**DOI:** 10.1155/2018/2474173

**Published:** 2018-10-31

**Authors:** Erkut Küçük, Uğur Yılmaz, Kürsad Ramazan Zor

**Affiliations:** ^1^Ophthalmology Department, Niğde Ömer Halisdemir University, Faculty of Medicine, 51240 Niğde, Turkey; ^2^Ophthalmology Department, Pamukkale University Faculty of Medicine, 20160 Denizli, Turkey

## Abstract

**Purpose:**

In this study, we evaluated corneal epithelial integrity and tear film parameters in patients with inflamed pinguecula and compared these findings with their fellow eyes and with healthy controls.

**Methods:**

We evaluated the fluorescein staining properties and performed the tear break-up time (TBUT) test and Schirmer 2 test (ST2) measurements of 32 patients who had symptomatic unilateral inflamed pinguecula and compared the results with their fellow eyes and also with an age- and sex-matched control group.

**Results:**

Twenty-three eyes (72%) in the inflamed pinguecula group and 1 eye (3.1%) in the fellow eyes group had punctate epithelial staining (PES) or epithelial defect on the nasal cornea (*p* < 0.001). There was no PES or epithelial defect in the control group. Eyes with inflamed pinguecula (*n* = 32) had lower TBUT and ST2 values compared to the control group (*n =* 32) (*p* < 0.001 for both). Fellow eyes (*n =* 32) also had lower TBUT and ST2 values compared to the control group (*p*=0.003 for both). There was no difference in the TBUT and ST2 results between the eyes with inflamed pinguecula and fellow eyes (*p*=0.286 and *p*=0.951, respectively).

**Conclusion:**

A high percentage of eyes with inflamed pinguecula had nasal corneal epithelial staining or epithelial defect. We also found lower TBUT and ST2 results in eyes with inflamed pinguecula and the fellow eyes compared to the control group. These findings may be important in pathogenesis of pinguecula and pterygium and also in uncovering their relation.

## 1. Introduction

Pinguecula is a yellowish elevated mass commonly located on the nasal bulbar conjunctiva close to the limbus [1]. Its prevalence increases with age, and ultraviolet radiation (UVR) is a risk factor in its pathogenesis [2, 3]. Male gender and diabetes mellitus are also reported risk factors [4]. Histological studies reported abnormal differentiation and squamous metaplasia of the conjunctival epithelium, exaggeration and distortion in the production of elastic fibers, and abnormality of their organization in the subepithelial connective tissue [5–7]. It was reported that 22.5% to 70.1% of the population has pinguecula [4, 8]. This heterogeneity in the prevalence may be due to differences in age, geographic location, and ethnicity of participants. Pinguecula may be inflamed, causing hyperemia, pain, and foreign body sensation.

Pterygium is a triangular growth of conjunctival fibrovascular tissue onto the cornea, usually located at the nasal cornea. Its prevalence is lower than that of pinguecula. It can cause decreased visual acuity, irritation, and pain due to inflammation and cosmetic problems. Although surgery is effective in its treatment, the risk of recurrence is still an important problem. Ultraviolet radiation (UVR) is thought to be a factor in the development of both pinguecula and pterygium. It is hypothesized that UVR causes conjunctival degeneration and the formation of pinguecula. With increased exposure, corneal epithelial and stem cells may be affected and lead to the formation of pterygium [9, 10]. But it is still unknown if pinguecula is a precursor of pterygium or if so, what causes its progress to pterygium.

Several studies reported abnormalities of tear function tests in pinguecula patients [2, 11]. The abnormality of the tear film and mechanical trauma may cause inflammation of pinguecula [12]. Inflamed pinguecula has attracted little attention in the ophthalmic community. In this study, we investigated the fluorescein staining properties and tear film parameters in patients with inflamed pinguecula. We also discussed the role of these parameters in the possible evolution of the inflamed pinguecula to pterygium.

## 2. Materials and Methods

This controlled multicenter study was performed in the Ophthalmology Department of Niğde Ömer Halisdemir University (Niğde, Turkey) and Ophthalmology Department of Pamukkale University Hospital (Denizli, Turkey). Both cities are located at the same latitude (38°), and they have the same distance from the equator. Denizli is located approximately 124 km from the Aegean Sea, and Niğde is located 130 km from the Mediterranean Sea. Although regional differences can exist, these two cities show similar climatic characteristics. Thirty-two consecutive patients who applied to these clinics between July 2017 and September 2017 and had symptomatic unilateral inflamed pinguecula were included. Twelve of these patients were from Pamukkale University Hospital and 20 from Niğde Ömer Halisdemir University Ophthalmology Department. Symptomatic inflamed pinguecula was described as a combination of vascular congestion and hyperemia of the pinguecula and adjacent conjunctiva in biomicroscopic examination together with patients' description of a recent increase in ocular redness and one or more of the following symptoms: photophobia, pain, foreign-body sensation, discomfort, and tearing. Two independent experienced ophthalmologists (EK and UY) diagnosed the patients for inclusion criteria. A control group (*n* = 32) was formed from age-matched individuals that did not have any ophthalmic disease other than refractive problems. Subjects who had corneal pathologies, allergic conditions, previous corneal and/or conjunctival surgery, meibomian gland dysfunction, active ocular infection, and contact lens users were excluded. All participants underwent complete ophthalmologic examination. To ensure reproductivity, all patients diagnosed with inflamed pinguecula were reexamined, and tests of the tear function were performed on the following day in the morning in the ophthalmologists' dimly lit examination room. Corneal staining properties were evaluated using fluorescein sodium solution 2% (Fluorescite®; Alcon Laboratories, Inc., Fort Worth, Texas 76134, USA). For TBUT test measurements, a drop of 2% fluorescein solution was applied to the lateral inferior fornix. The patient was asked to blink several times for uniform distribution of fluorescein and then instructed to look ahead without blinking. The time from the last blink to the appearance of the first dry spot on the cornea was recorded using the cobalt blue filter of the biomicroscope and a stopwatch. Three consecutive measurements were made, and the mean of measurements was recorded. Thirty minutes later, in the dimly lit examining room, a topical anesthetic agent proparacaine hydrochloride 0.5% drop (Alcaine®; Alcon, Fort Worth, TX) was applied to the inferior fornix, and three minutes later, a standard Schirmer test filter strip (Bio Schirmer®; Bio-Tech Vision Care, Ahmedabad, Gujarat, India) was inserted into the lateral inferior fornix at the junction of the middle and lateral thirds of the lower eyelid, taking care not to touch cornea. The patient was asked to keep eyes open and blink as necessary. After five minutes, the filter strip was removed and wetting was recorded. This study was performed according to the tenets of Declaration of Helsinki, and the study received approval from Pamukkale University Ethics Committee. Written informed consent and verbal informed consent were taken from patients and controls.

Statistical analysis was performed using SPSS version 20.0 (IBM Corporation, Armonk, NY). Test results were expressed as mean ± standard deviation (SD). The distribution of the variables was tested using the Kolmogorov–Smirnov test. The chi-square test was used to compare groups for gender and nasal corneal epithelial staining. Independent-samples *T* test was used to compare the groups for age. For BUT and ST2 values, the Kruskal–Wallis one-way test was used to test the difference among groups and Mann–Whitney *U* test was used to compare groups. In all analyses, *p* values <0.05 were considered as statistically significant.

## 3. Results

**Figure 1:**
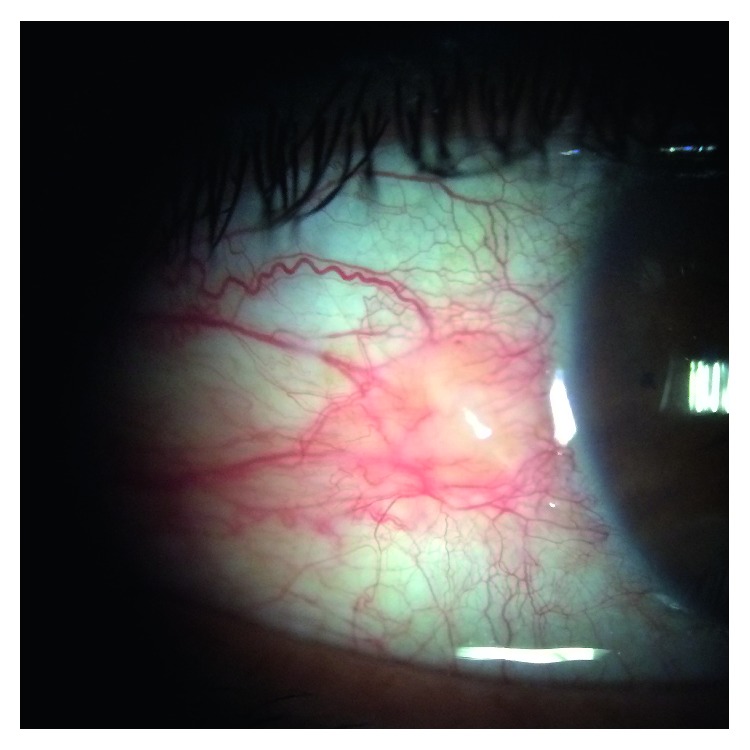
An inflamed pinguecula.

There was no significant difference in age and gender between inflamed pinguecula and control groups (*p*=0.862 and *p*=0.794, respectively) (Table 1). Thirty-two eyes of 32 patients had inflamed pinguecula. All inflamed pingueculae were on the nasal conjunctiva (Figure 1). There were pinguecula in 13 (40 %) and pterygium in 3 (9%) of the fellow eyes (*n* = 32). There was no pinguecula or pterygium in the control group.

**Figure 2:**
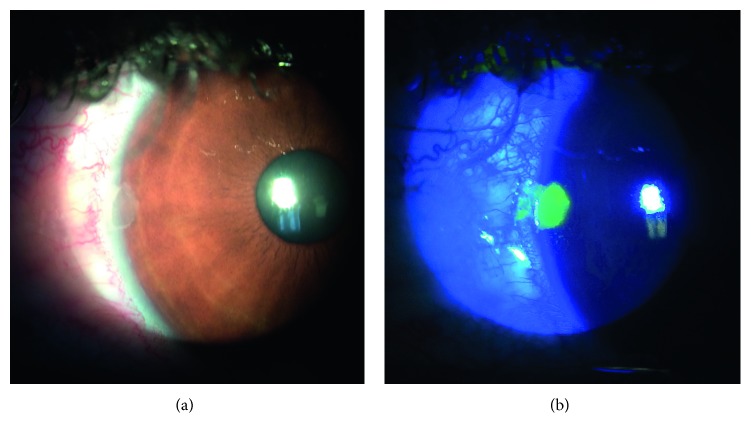
(a) Epithelial defect and (b) fluorescent staining in a patient with inflamed pinguecula.

Twenty-three eyes (72%) had punctate epithelial staining (PES) or epithelial defect on the nasal cornea in eyes with inflamed pinguecula (Figures 2(a) and 2(b)). There was one eye (3.1%) with corneal PES in the fellow eyes group. The difference was statistically significant (*p* < 0.001). There was no corneal PES or epithelial defect in the control group.

The mean values of TBUT tests of eyes with inflamed pinguecula, fellow eyes, and control eyes were 8.1 ± 3.9 s, 9.3 ± 4.3 s, and 13.5 ± 4.9 s, respectively (Table 2). The eyes with inflamed pinguecula had significantly lower TBUT values compared to the control group (*p* < 0.001). Fellow eyes also had lower TBUT values than the control group (*p*=0.003). There was no significant difference in the TBUT results between eyes with inflamed pinguecula and fellow eyes (*p*=0.286). The mean values of ST2 results of eyes with inflamed pinguecula, fellow eyes, and control eyes were 11.6 ± 5.1 s, 11.6 ± 5.3 s, and 17.6 ± 7.8 s, respectively. The eyes with inflamed pinguecula had significantly lower ST2 values compared to the control group (*p* < 0.001). Fellow eyes also had lower ST2 values than the control group (*p*=0.003). There was no significant difference in the ST2 results between the eyes with inflamed pinguecula and fellow eyes (*p*=0.951).

## 4. Discussion

Pinguecula is a common disease of the conjunctiva whose exact etiology is unknown. UVR is reported to be an important factor [2, 3]. Fluorescein is a diagnostic dye commonly used in ophthalmic practice. Although the underlying cellular mechanism of corneal staining is incompletely understood, fluorescein staining of the ocular surface is a common diagnostic feature of ocular diseases, and it is frequently used to assess ocular surface integrity, particularly the cornea [13, 14]. A high rate of nasal corneal PES or epithelial defect was present in the inflamed pinguecula group compared to fellow eyes and control group in our study. This finding was not reported in previous studies. We could not find reports regarding the fluorescein staining of the nasal cornea in pinguecula patients in our literature review. The pathogenesis of this staining may be similar to dellen formation in which corneal thinning occurs usually close to limbus due to reduced tear film spread over a focal corneal area and is usually associated with an adjacent focal conjunctival or corneal elevation. Reduced tear break-up time was also reported to be associated with dellen formation [15]. In dry eye patients, corneal fluorescein staining usually occurs symmetrically on the corneal surface without a predilection for a specific part [16]. Our study suggests that an inflamed and elevated pinguecula may affect the distribution of the tear film and cause a desiccated epithelium in the nasal cornea close to the limbus. Also, impaired tear function evidenced by lower TBUT and ST2 results in these patients may aggravate this situation. These factors together may cause epithelial cell damage and staining in the nasal cornea. There may be other effects of inflammation on the nasal corneal epithelium other than affecting tear film spread since previous reports on pinguecula without inflammation did not report nasal corneal fluorescein staining. The inflammatory cells and mediators may cause epithelial cell damage or may affect the epithelial healing in inflamed pinguecula patients.

Oğuz et al found that eyes with pinguecula have significantly lower TBUT values compared to the healthy controls [11]. Schirmer 1 test (ST1) results were not significantly different between the eyes with pinguecula and control group in their study. Dong et al. found that TBUT values improved after pinguecula excision, but ST1 results did not change [5]. Both TBUT and ST2 results were significantly lower in the eyes with inflamed pinguecula and fellow eyes compared to the control group in our study. TBUT measurements have inherent variability, and taking multiple readings and averaging the results is one way of improving repeatability [17]. Therefore, we used averaging the multiple readings in our study. Similar to these studies, TBUT values were also lower in our study. But unlike them, we also found lower ST2 results. This may be due to difference in study population since we investigated only patients with inflamed pinguecula. The results of our study indicate that both tear film stability and tear production were affected in patients with inflamed pinguecula. Balogun and coworkers compared the TBUT values of pterygium and pinguecula patients and healthy controls. The mean TBUT values were not significantly different between pinguecula group and healthy controls [18]. The inclusion of only inflamed pinguecula patients in our study and the differences in the geographic location and age of the participants may explain the different findings of Bolagun's study and the present one.

To understand whether inflamed pinguecula causes abnormalities of tear film or tear film abnormalities cause inflammation of the pinguecula, we compared the test results of these patients with those of the fellow eyes. We found that the TBUT and ST2 results are not significantly different between the eyes with inflamed pinguecula and fellow eyes. Fellow eyes also had abnormalities of the tear film function, and nearly 50% of these eyes had uninflamed pinguecula (40%) or pterygium (9%). Considering one of our diagnostic criteria of inflamed pinguecula “patients description of a recent increase in ocular redness” together with these results of the fellow eyes, we think that abnormality of the tear film may be present before the inflammation of pinguecula similar to current results of fellow eyes. Our study suggests that impaired tear film together with mechanical irritation of this elevated tissue makes pinguecula prone to inflammation.

Pterygium is a triangular growth of conjunctival fibrovascular tissue onto the cornea. Specific stimulus leading to pterygium formation is still unknown [19]. Although there are similarities in the pathogenesis and histopathological findings of these two ocular surface diseases, it is still unknown if pinguecula is a precursor of pterygium and if so, what causes it to progress to pterygium [1, 6]. Dong et al. reported that abnormal epithelial differentiation is present in pinguecula tissue and that pinguecula epithelium has proliferative capacity exhibiting characteristics of squamous proliferative diseases [5]. There are also several reports indicating the role of inflammatory cytokines and growth factors (GFs) in the pathogenesis of pterygium [19–21]. These GFs and cytokines are also important in the normal corneal wound healing and overexpressed in pterygia. Interleukin-1 and epidermal growth factor were reported to be important, and they have an additive effect on corneal epithelial cell migration in corneal epithelial wounds [22]. Epidermal growth factor was also shown to induce cell migration in pterygium epithelium and fibroblasts [20]. Kim et al. emphasized the importance of myofibroblasts in pterygium formation [23]. They stated that pterygium may be a product of an exaggerated repair process after injury to the ocular surface and prolonged inflammation leading to tissue damage and fibrosis. They also emphasized the importance of stromal cell-derived factor-1 and transforming growth factor-beta with other GFs and inflammatory mediators in the activation of pterygium fibroblasts. These studies mainly emphasize the importance of inflammatory cytokines and GFs in the pterygium formation and that the pterygium may be an exaggerated repair process.

Archila and Arenas stated that exposure to chronic solar radiation causes alteration of conjunctival stroma and leads to pinguecula formation. This causes disruption of tear film and an area of dryness which results in drying of conjunctiva and formation of microulcers on the epithelium. Then, as a part of protective changes, conjunctiva tries to cover erosion and leads to pterygium formation [24]. Based on the literature, our results suggest that abnormal tear film and improper lubrication together with ocular surface irregularity due to pinguecula may cause inflammation of the pinguecula, and these factors cause epithelial defects on the nasal cornea. Inflammation and corneal epithelial damage may cause release of GFs and cytokines which act together to close the wound and relieve the inflammation in these patients. UVR was reported to cause limbal stem cell failure on the nasal cornea [10]. When corneal healing does not occur properly due to limbal stem cell failure, a prolonged inflammatory response and exaggerated wound healing process may occur, and these mediators act on pinguecula epithelium and stroma, leading to proliferation towards the nasal cornea to close the wound. Our study suggests that nasal corneal epithelial damage in inflamed pinguecula patients may be a stimulus for exaggerated wound repair causing the release of GFs leading to growth of conjunctival epithelium onto the cornea. Inflamed pinguecula patients with impaired ocular surface lubrication and nasal corneal epithelial defects may be a subgroup of pinguecula patients who have a propensity to progress to pterygium.

Our study is a cross-sectional study, and it is a limitation of our study. Another limitation is that we did not perform histologic or cytologic examination. The diagnosis of pinguecula is mostly clinical, and due to typical appearance, diagnosis of pinguecula is usually easy but sometimes other pathologies can mimic pinguecula.

In conclusion, to our knowledge, this is the first study evaluating corneal staining properties and tear functions in inflamed pinguecula patients. In a high percentage of inflamed pinguecula groups, we found nasal corneal epithelial staining or epithelial defect. We found lower TBUT and ST2 results in this group. These findings may be important in uncovering the relation of pinguecula and pterygium and also in their pathogenesis. Inflammation, corneal epithelial integrity, and impaired tear film parameters may be important factors in the evolution of the pinguecula to pterygium.

## Figures and Tables

**Table 1:** Demographic characteristics of groups.

		Inflamed pinguecula group (*n*=32)	Control group (*n*=32)	*p*
Age (years) (mean ± SD)		32.78 ± 10.35	32.31 ± 11.07	**0.862** ^**a**^
Sex	Female, *n* (%)	21 (65.6%)	20 (62.5%)	**0.794** ^**b**^
	Male, *n* (%)	11 (34.4%)	12 (37.5%)	

^a^Independent-samples *T* test; ^b^chi-square test; *p* value <0.05 is statistically significant.

**Table 2:** Schirmer 2 and TBUT test results of the groups.

		Patient eyes with inflamed pinguecula (*n* = 32) (Group 1)	Patient eyes without inflamed pinguecula (*n* = 32) (Group 2)	Control eyes (*n =* 32) (Group 3)	*p* ^*∗*^	*p* ^#^ for intergroup comparisons		
						Groups 1 vs 2	Groups 1 vs 3	Groups 2 vs 3
BUT (s)	Mean ± SD	8.1 ± 3.9	9.3 ± 4.3	13.5 ± 4.9	<0.005	0.286	<0.001	0.003
	Median	8.0	8.0	14.0				
	Range	3–19	3–18	4–25				

ST2 (mm)	Mean ± SD	11.6 ± 5.1	11.6 ± 5.3	17.6 ± 7.8	<0.005	0.951	<0.001	0.003
	Median	11.0	10.5	20.0				
	Range	3–22	2–21	4–30				

^*∗*^ *p* value for comparison among three groups (Kruskal–Wallis one-way test). ^#^*p* values for intergroup comparisons (Mann–Whitney *U* test). *p* value <0.05 is statistically significant.

## Data Availability

The data used to support the findings of this study are available from the corresponding author upon request.
